# Compound Shenma Jingfu granule alleviates cerebral ischemia via HIF-1α-mediated promotion of angiogenesis

**DOI:** 10.1186/s13020-024-00926-w

**Published:** 2024-04-10

**Authors:** Ruihua He, Yi Xu, Jingxue Liu, Jing Liu, Jing Chen, Xufang Wang, Lei Qiu, Jin Huang

**Affiliations:** 1 Department of Pharmacy, Yueyang Hospital of Integrated Traditional Chinese and Western Medicine, Shanghai University of Traditional Chinese Medicine, Shanghai, 200083 China; 2 Department of Pharmacy, Second Affiliated Hospital of Naval Medical University, Shanghai, 200003 China; 3 College of Pharmacy, Navy Medical University, Shanghai, 200433 China

**Keywords:** Shenma Jingfu granule, Cerebral ischemia, HIF-1 signaling pathway, Angiogenesis

## Abstract

**Background:**

Shenma Jingfu Granule, a traditional Chinese medicine formula, has been used clinically for the treatment of cerebral circulation insufficiency. However, the mechanism involved in alleviating cerebral ischemia has not yet been fully elucidated.

**Methods:**

An integrated approach involving network pharmacology and transcriptomics was utilized to clarify the potential mechanisms of SMJF Granule. Molecular docking and surface plasmon resonance (SPR) were employed to identify potential targets and ingredients of SMJF Granule. The anti-CI effect of SMJF Granule was determined on the middle cerebral artery occlusion (MCAO) model by using hematoxylin–eosin (H&E) and Nisslʼs staining, as well as triphenyl tetrazolium chloride (TTC) staining, and the potential targets involved in the mechanisms were validated by RT-qPCR and western blotting.

**Results:**

Integrated analysis revealed the mechanism of SMJF Granule intervening in CI injury might be related to the HIF-1 signaling pathway and angiogenesis. Molecular docking and SPR assays demonstrated robust binding interactions between key compounds like salvianolic acid A and naringenin with the core target HIF-1α protein. The experiment confirmed that SMJF Granule lowered neurological scores, diminished infarct volume, and alleviated histopathological changes in vivo. The possible mechanism of SMJF Granule was due to regulating HIF-1 pathway, which contributed to up-regulating expression of VEGF and vWF in the penumbral region, showing a significant promotion of angiogenesis.

**Conclusion:**

SMJF Granule promoted angiogenesis through HIF-1α pathway, thereby alleviating cerebral ischemia injury. In addition, our findings provide some evidence that SMJF Granule is a candidate compound for further investigation in treating CI in the clinical.

**Graphical Abstract:**

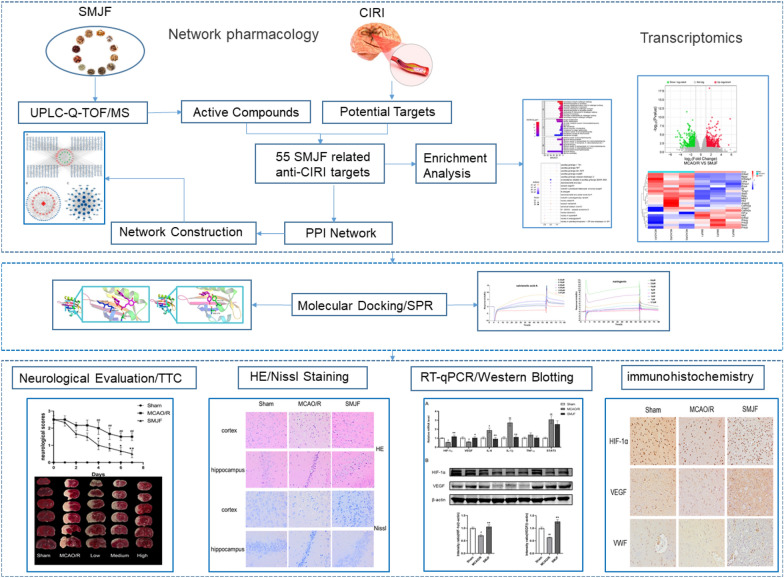

**Supplementary Information:**

The online version contains supplementary material available at 10.1186/s13020-024-00926-w.

## Introduction

Stroke, encompassing ischemic and hemorrhagic categories, is the leading global cause of death and disability [1]. In the past thirty years, the incidence of cerebral ischemia has been increasing [2], especially among those under 55 [3], underscoring the need for more effective therapeutic and preventive strategies. Cerebral ischemia injury involves initial injury during ischemia and subsequent reperfusion-induced damage [4], driven by mitochondrial dysfunction, elevated oxidative stress/reactive oxygen species (ROS), blood–brain barrier (BBB) disruption, inflammation, and apoptosis [5]. Consequently, combining reperfusion with anti-inflammatory or neuroprotective agents holds promise for acute ischemic stroke treatment [6]. Nonetheless, clinical neuroprotective agents like nimodipine [7] and edaravone [8] often target single pathways. At the same time, the development of clinically effective agents for stroke is still an urgent task.

The compound prescription of traditional Chinese medicine (TCM), which possesses a spectrum of therapeutic properties, including antioxidative, anti-inflammatory, anti-apoptotic, neuroprotective, and vascular protective effects, has unique advantages in medical practice. These attributes position them as promising candidates for ischemic stroke treatment, holding significant potential for stroke therapy [9]. Nevertheless, the mechanisms of action of such compounds require further elucidation, warranting ongoing research.

One such candidate is the SMJF Granule, a well-established TCM prescription with a rich clinical history. Comprising twelve herbs (as outlined in Table 1), SMJF Granule is renowned for its efficacy in enhancing blood circulation, clearing collaterals, calming the mind, rejuvenating the brain, and strengthening muscles and bones. Clinically, it finds application in treating conditions like cervical spondylosis, insufficient cerebral blood flow, vertigo, and nocturnal insomnia [10]. Despite its widespread use, the active constituents and underlying therapeutic mechanisms responsible for SMJF's effects remain largely uncharted territory, necessitating thorough investigation.

**Table 1:** Chinese herbs of SMJF Granule

Chinese name	Botanical name	Part used
Shouwuteng	*Reynoutria multiflora* (Thunb.) Moldenke	Vine and stem
Danshen	*Salvia miltiorrhiza* Bunge	Root
Shanzhuyu	*Cornus officinalis* Siebold & Zucc	Fruit
Zhizi	*Gardenia jasminoides* J.Ellis	Fruit
Jili	*Tribulus terrestris* L	Fruit
Sangjisheng	*Taxillus chinensis* (DC.) Danser	Leaf and stem
Xuduan	*Dipsacus asper* Wall. ex DC	Root
Duzhong	*Eucommia ulmoides* Oliv	Bark
Tianma	*Gastrodia elata* Blume	Tuber
Danggui	*Angelica sinensis* (Oliv.) Diels	Root
Chuanxiong	*Ligusticum striatum* DC	rhizome
Chenpi	*Citrus aurantium f. deliciosa* (Ten.) M.Hiroe	peel

In recent years, network pharmacology has played a significant role in advancing our understanding of the fundamental composition and functional mechanisms of TCM. Furthermore, it has been instrumental in uncovering the synergistic interactions among various ingredients, driving the modernization of traditional medicine [11]. The efficacy of TCM relies on the interplay among its chemical constituents. While TCM contains multiple ingredients, only those that effectively enter the bloodstream can exert specific effects [12]. Therefore, the integration of UPLC-Q-TOF-MS and network pharmacology analysis is widely used to identify active ingredients in compound drugs. This approach stands as a robust method for unraveling therapeutic mechanisms and pinpointing potential targets for novel drug development.

**Fig. 1:**
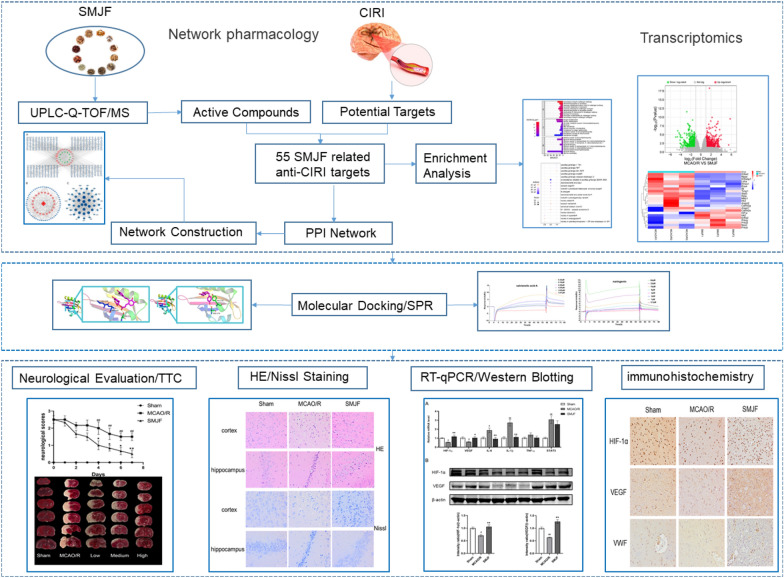
Strategy of the research method for anti-CI mechanism of SMJF

In this study, UPLC-Q-TOF–MS was utilized to identify the key constituents present in SMJF Granule, both in the bloodstream and within ischemic lesions. Employing network pharmacology, we elucidated the intricate interactions within the "ingredient-target-disease" paradigm, providing a comprehensive insight into the relationship between the compound and CI. This holistic and systemic approach aligns seamlessly with the principles of TCM. Furthermore, we conducted experimental validation using an animal model of MCAO/R treated with SMJF Granule to delve deeper into their efficacy and underlying mechanisms (Fig. 1).

## Materials and methods

### Drugs and reagents

Shenma Jingfu Granule (Approval No: HYZZ 205050324) was provided by Yueyang Hospital, Shanghai University of Traditional Chinese Medicine. Anti-VEGF Polyclonal Antibody (K009531P) was purchased from Solarbio (Beijing, China). Anti-HIF-1 Alpha antibody (bs-20399R) was from Bioss (Beijing, China). Anti-vWF antibody (PB9273) was purchased from Boster (Wuhan, China). Standards used for SPR analysis were provided by Standard Technology (Shanghai, China).

The World Flora Online (http://www.worldfloraonline.org) was used to verify the complete plant names on December 8, 2023.

### Chemical identification and profiling by UPLC-Q-TOF -MS

The chemical composition of the SMJF Granule was analyzed through UPLC-Q-TOF–MS. In brief, a Waters ACQUITY UPLC HSS T3 column (2.1 × 100 mm, 1.8 µm) was utilized for gradient elution. As the mobile phase, distilled water was mixed with 0.1% formic acid solution and 0.1% acetonitrile solution. Flow rate: 0.3 mL/min. This experiment was conducted with a 30 °C column temperature, and the injection volume was 2 μL. The detection wavelength varied from 190 to 400 nm. Mass spectrometry detection was performed in ESI negative/positive ion mode. Ion Source Gas 1/2: 50 psi; Ion Source Temperature: 500 °C; Ion Spray Voltage Floating: − 4500/5000 V; Declustering Potential: 100 V; Collision Energy: ± 40 eV; TOF mass range: 50 ~ 1700; MS/MS mass range: 50 ~ 1250. Qualitative analysis of SMJF Granule was performed, resulting in total ion flow diagrams in both positive and negative modes under the mentioned conditions.

Five rats were given oral suspensions (1.2 g/kg, seven times the clinical equivalent dose) of SMJF Granule twice daily for five consecutive days to determine the absorption of the ingredients in plasma and brain. On the final day, rats were euthanized, and samples of plasma and brain tissue were obtained and analyzed by UPLC-Q-TOF–MS for the absorbed ingredients of SMJF Granule.

### Prediction of SMJF Granule candidate targets in CI

The potential targets of absorbed ingredients in blood and brain were predicted by the PharmMapper (http://www.lilab-ecust.cn/pharmmapper/), Swiss Target Prediction (http://www.swisstargetprediction.ch/) and HERB (http://herb.ac.cn/) databases. Disease targets related to cerebral ischemia were collected by searching DisGeNET (http://www.disgenet.org/web/ DisGeNET/menu/home), Therapeutic Target Database (TTD) (https://db.idrblab.org/ttd/), and OMIM database (https://omim.org/) using the keywords “cerebral ischemia” and “ischemia stroke”. In addition, the Venn diagram was used to screen the intersection targets of the absorbed ingredients of SMJF Granule and cerebral ischemia. To visualize the network of SMJF Granule twelve herbs-ingredients-targets, the Cytoscape software (version 3.8.0) was employed.

### Protein–protein interaction (PPI) network and functional enrichment analysis

Understanding the complex relationship between SMJF Granule and cerebral ischemia targets is challenging due to intricate biological processes involving multiple proteins. To address this, the overlapping targets were then uploaded to the STRING database (https://string-db.org/) with “Homo sapiens” as the species, and only those targets with interaction values exceeding 0.4 were considered for further analysis. The resulting PPI network was visualized in Cytoscape. To understand the roles of the targets of SMJF Granule active ingredient in particular signal pathways, GO enrichment analysis and KEGG pathways with modified values of p less than 0.05 for the potential targets of SMJF Granule in the treatment of cerebral ischemic were obtained. The GO biological process, cellular analysis, molecular function, and KEGG pathway dot plot of core targets were plotted by https://www.bioinformatics.com.cn.

### Transcriptome analysis

The total RNA from the penumbra tissues of rat brains was extracted by the Trizol reagent kit with the determination of their integrity and concentration. Subsequently, the RNA was randomly fragmented, followed by the synthesis of double-stranded cDNA. The construction of cDNA libraries involved end repair, poly(A) tailing, and sequencing adapter ligation, followed by PCR amplification. After library quantification, high-throughput sequencing of multiple samples was conducted using the Illumina HiSeq sequencing platform in paired-end mode. The FastQC software was used to conduct a quality control examination on preprocessed data. With STAR software, the preprocessed sequences were matched to the sequenced species' reference genome sequence. Subsequently, to determine the quantity of reads mapped to every gene, StringTie software was utilized. Gene length was used to compute each gene’s FPKM (fragments per kilobase per million) values and read counts which were then mapped to the respective genes. Differential expression analysis between different sample groups was conducted using DESeq2 software, with the criteria of |log2FC|≥ 1 and P ≤ 0.05 used to filter differentially expressed genes between the two groups.

### Molecular docking

For molecular docking in AutoDock 1.5.6 Vina, we selected the key compounds with the higher degrees in the SMJF Granule twelve herbs-ingredients-targets network and the HIF-1α protein. The 3D structure of the protein was downloaded from the RCSB PDB database (https://www.rcsb.org/), and the 2D structural compounds were obtained from the PubChem database (https://pubchem.ncbi.nlm.nih.gov/). The protein and compounds were preprocessed using PyMol 2.4.0 software. Molecular docking and binding affinity calculations were conducted by AutoDock Vina. The 3D structure of the complexes was visualized with PyMol to observe the interaction between the receptor protein and compounds, including hydrogen bonding.

### SPR analysis

In this study, the HIF-1α protein was chosen for SPR analysis based on network pharmacology. Standards were dissolved in DMSO to 20 mM. SPR assays were performed using a Biacore T200. Briefly, the protein was immobilized on a CM5 chip via EDC/NHS cross-linking. Standard compounds were diluted (0.01–128 µM) in 5% DMSO-phosphate buffered saline. Sensor surface stability was assessed by repeating the average concentration. Analytes were injected at 30 μL/min with 60 s binding and 120 s dissociation times. Biacore T200 evaluation software was used for affinity curve fitting, employing a 1:1 ratio steady-state affinity model. Meanwhile, kinetic parameters were calculated.

### Animal grouping and treatment

Male SPF-grade SD rats (Shanghai SLAC Laboratory Animal Co., Ltd; license number SCXK(Hu) 2022–0004) were kept in an observation room with a 12-h cycle of light and darkness, maintaining temperature (22–24 °C) and humidity (40–70%). During the period of adaptive feeding, the rats were provided with unrestricted access to sterile water and conventional laboratory chow for one week. Animal experiments were approved by Yueyang Laboratory Animal Center Animal Ethics Committee of Shanghai University of Traditional Chinese Medicine (YYLAC-2022-169).

Following a prior study, we induced cerebral ischemia with an MCAO/R model in rats. Anesthesia was administered via a 2% intraperitoneal sodium pentobarbital injection at 40 mg/kg. After the neck incision, we exposed and separated the left common carotid artery and vagus nerve. An appropriately sized nylon monofilament was placed into the internal carotid artery through the common carotid artery, followed by ligation to block the left middle cerebral artery's origin. After 2 h, we removed the monofilament for reperfusion.

Three groups of rats were assigned at random: sham, MCAO/R, and MCAO/R + SMJF Granule (1.2 g/kg) groups. Sham control rats underwent a similar surgical procedure without MCAO ligation. SMJF Granule was administered 24 h post-MCAO/R, twice daily for 7 days. Rats in the sham and MCAO/R group received an equivalent volume of saline.

### Neurobehavioral score

Neurological deficits in rats were evaluated in a single-blind fashion after reperfusion by Longa et al. [13]. The scoring criteria were as follows: 0 points: no nerve damage symptoms; 1 point: incomplete extension of the right forepaw; 2 points: rightward turn; 3 points: rightward collapse; 4 points: impaired walking and unconsciousness. Rats with scores of 1 to 3 were included in the results.

### Triphenyl tetrazolium chloride (TTC) staining

After the brain tissue was removed, it was immediately frozen at − 20 °C for 15 min. Coronal sections were then taken with an even thickness of 2 mm, resulting in 6 layers of sections. These sections were then incubated in a water bath protected from light with a configured 2% TTC staining solution at 37 °C for 30 min. To ensure uniform staining on both sides, the sections were turned every 5 min. After staining, 4% paraformaldehyde was added for fixation. The percentage of brain infarct volume was calculated by the Image J software. Percentage of infarct volume (%) = total infarct volume/whole brain volume*100%.

### HE and Nissl staining

To examine the pathological alterations, the brain tissue specimen was underwent fixation using 4% paraformaldehyde. Then the sample was sliced to a 3 μm thick paraffin section for HE and Nissl staining. Microscopic observation of stained cerebral sections was then performed, facilitating the study of morphological transformations within the cerebral cortex and the hippocampal CA1 region.

### Real-time quantitative PCR analysis

RNA was extracted and purified from the ischemic penumbra using the kit according to the Beyotime manufacturer's instructions. The mRNA expression of candidate targets was assessed by real-time quantitative PCR with fluorescence detection. The primer sequences used in this study are shown in Table 2. The relative expression levels of target genes were normalized to that of β-actin using the 2^−ΔΔCt^ method.

**Table 2:** Sequences of the primers used for RT-qPCR

Primer name	Forward primer (5′–3′)	Reverse primer (5′–3′)
*HIF-1α*	TACTGATTGCATCTCCACCTTCTAC	CTGCTCCATTCCATCCTGTTC
*VEGF*	CGCCAAGCCCGGAAGATTAG	CCAGGGATGGGTTTGTCGTG
*IL-6*	TCCAGTTGCCTTCTTGGGAC	GTGTAATTAAGCCTCCGACTTG
*IL-1β*	GAGCTTCAGGAAGGCAGTGTCAC	TCCACGGGCAAGACATAGGTAGC
*TNF-α*	AGATGTGGAACTGGCAGAGG	CCCATTTGGGAACTTCTCCT
*STAT3*	TCCTGCTGCGGTTCAGTGAG	GCTGCTGCTTGGTATATGGTTCTAC
*β-Actin*	TGTTACCAACTGGGACGACA	GGGGTGTTGAAGGTCTCAAA

### Western blotting

The total proteins of ischemic penumbra tissue were extracted, and measured through a BCA protein assay. Afterward, equal amounts of protein were electrophoretically separated by SDS-PAGE and immobilized on nitrocellulose membranes (Millipore Corporation, Bedford, USA), which were subsequently immersed in 5% skimmed milk at room temperature for 2 h. Next, the primary antibodies were incubated with the membranes overnight at 4 °C. Subsequently, the secondary antibodies were added and incubated at room temperature for 1 h. The Odyssey system (LICOR, Lincoln, Nebraska) was utilized for visualization. The quantification of protein band densities was performed using Image J software.

### Immunohistochemical staining

The infarcted cerebral hemispheres were fixed in 4% paraformaldehyde for 48 h, paraffin-embedded, and cut into 3 μm sections. Sections were deparaffinized, hydrated, blocked with goat serum for 1 h, and incubated overnight at 4 °C with diluted anti-HIF-1α, vWF, or VEGF antibodies (1:100). After washing with PBST, secondary antibodies were added for 30 min at room temperature, followed by DAB chromogenic staining. Nuclei were stained with hematoxylin for 2 min, and positive DAB expression was observed as brownish-yellow. Slides were mounted with neutral resins after drying. Eventually, sections were viewed and photographed under an inverted optical microscope (Olympus).

### Statistical analysis

Statistical analysis was conducted using GraphPad Prism 8.0 software. The mean ± SEM was used to express the data. Differences between groups were assessed using one-way ANOVA, with a p-value of less than 0.05 considered statistically significant.

## Results

### SMJF granule active ingredients and target screening

**Fig. 2:**
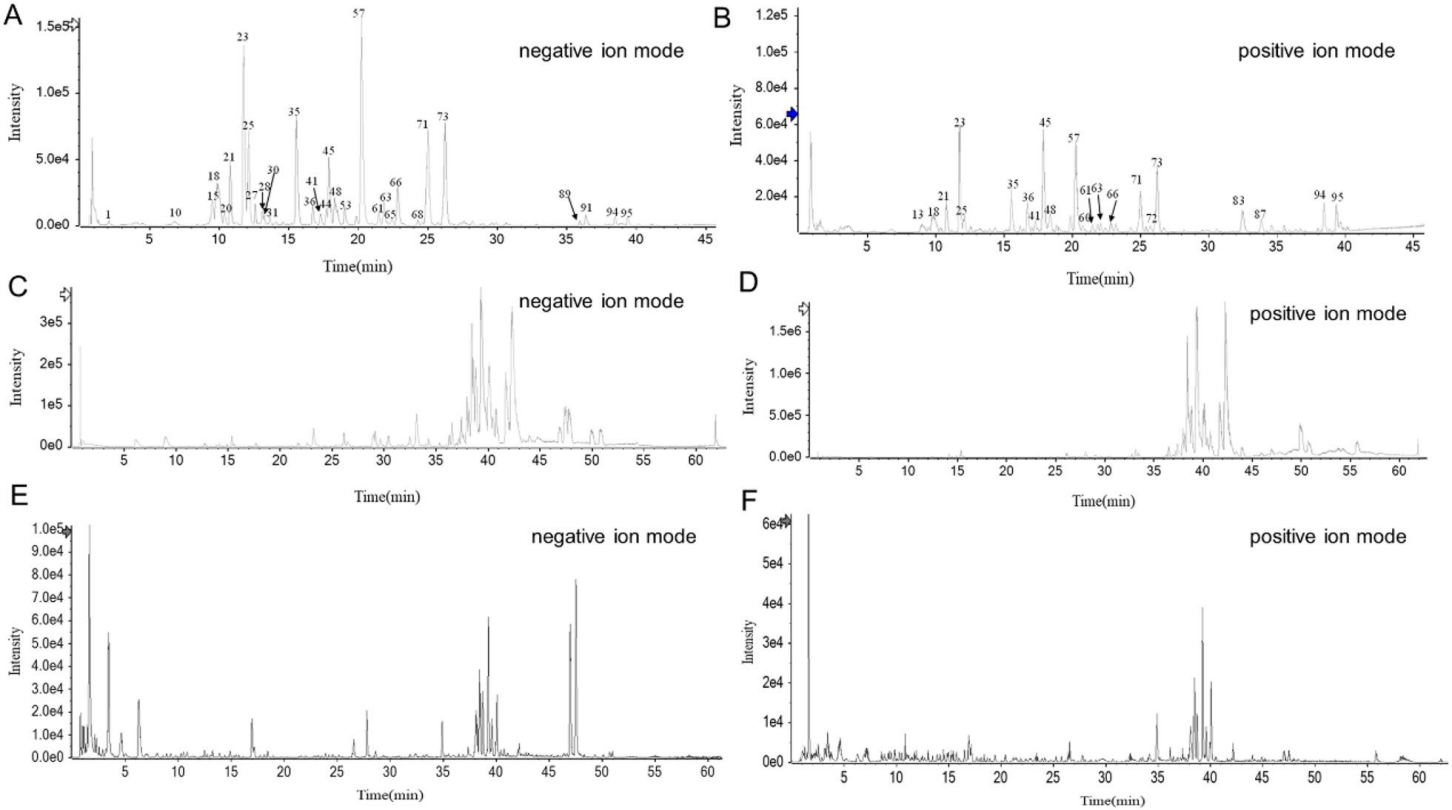
SMJF Granule was analyzed by UPLC-Q-TOF–MS. **A**, **B** UPLC-HRMS base peak ion chromatogram (BPC) of SMJF Granule sample. **C**, **D** UPLC-HRMS BPC of serum samples. **E**, **F** UPLC-HRMS BPC of brain tissue samples

**Fig. 3:**
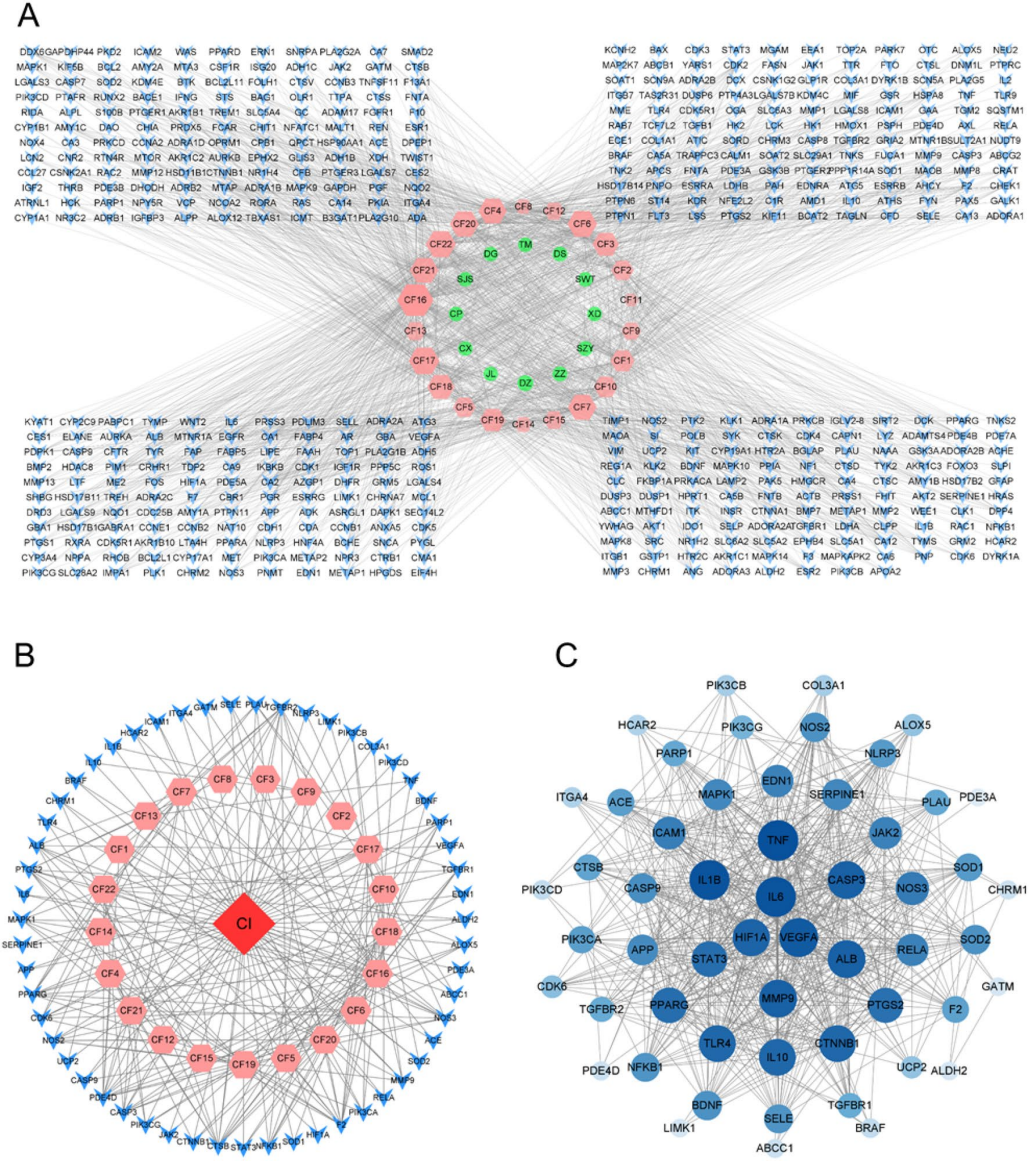
Target network analysis.** A** The SMJF Granule twelve herbs-ingredients-target network. **B** The ingredients-targets-CI network. **C** The PPI network of SMJF Granule potential targets with 55 nodes and 633 edges. The green node represents twelve herbs in SMJF Granule, the pink node represents ingredients, the red node represents disease, and the blue node represents potential targets

To investigate the active ingredients of SMJF Granule, UPLC-Q-TOF-MS technology was used to identify 99 ingredients (Fig. 2 and Additional file 1: Table S1). Further investigation was conducted on 22 compounds (Table 3) which were detected in blood and brain tissue samples after oral administration of SMJF Granule. Subsequently, 503 potential targets of SMJF Granule ingredients absorbed in the blood and brain were collected through the PharmMapper, Swiss Target Prediction, and HERB databases. An herbs-ingredients-targets network (Fig. 3A) and an ingredients-targets-disease (CI) network (Fig. 3B) were constructed. Then, an Analyze Network plugin was used to extract the most prominent compounds based on their degree, including salvianolic acid A, naringenin, pinoresinol diglucoside, loganin, and cryptotanshinone, et al. The results indicated that SMJF Granule might exert its efficacy through different ingredients and targets. Based on DisGeNET, TTD, and OMIM databases, 324 targets for cerebral ischemia were obtained, and 55 common genes were subsequently identified as potential targets of SMJF Granule against CI.

**Table 3:** Enriched ingredients of SMJF Granule in serum and brain

NO	Identification	CAS	Formula	Enrichment	Classification
CF1	Geniposidic acid	27741–01-1	C_16_H_22_O_10_	Serum, Brain	Zhizi/Duzhong
CF2	Shanzhiside methyl ester	64421–28-9	C_17_H_26_O_11_	Serum, Brain	Zhizi/Shanzhuyu
CF3	Genipin 1-gentiobioside	29307–60-6	C_23_H_34_O_15_	Serum	Zhizi/Duzhong
CF4	Geniposide	27745–20-6	C_17_H_24_O_10_	Serum	Zhizi/Duzhong
CF5	Sweroside	14215–86-2	C_16_H_22_O_9_	Serum	Xuduan/Shanzhuyu
CF6	Loganin	18524–94-2	C_17_H_26_O_10_	Serum	Xuduan/Shanzhuyu
CF7	2,3,5,4'-Tetrahydroxystilbene 2-O-glucoside	82373–94-2	C_20_H_22_O_9_	Serum	Shouwuteng
CF8	Eucommiol	55930–44-4	C_9_H_16_O_4_	Serum, Brain	Duzhong
CF9	Protocatechuic acid	99–50-3	C_7_H_6_O_4_	Serum, Brain	Danshen, Duzhong
CF10	danshensu	76822–21-4	C_9_H_10_O_5_	Serum, Brain	Danshen
CF11	p-Hydroxybenzyl alcohol	623–05-2	C_7_H_8_O_2_	Serum	Tianma
CF12	Ferulic acid	1135–24-6	C_10_H_10_O_4_	Serum	Duzhong/Danggui/Sangjisheng/Chenpi
CF13	Caffeic acid	331–39-5	C_9_H_8_O_4_	Serum	Duzhong/Chuanxiong
CF14	Genipin	6902–77-8	C_11_H_14_O_5_	Serum	Duzhong/Zhizi
CF15	3-hydroxycinnamic acid	588–30-7	C_9_H_8_O_3_	Serum	Duzhong
CF16	Salvianolic acid A	96574–01-5	C_26_H_22_O_10_	Serum	Danshen
CF17	Naringenin	480–41-1	C_15_H_12_O_5_	Serum	Chenpi
CF18	Emodin	518–82-1	C_15_H_10_O_5_	Serum	Shouwuteng/Jili
CF19	Pinoresinol	487–36-5	C_20_H_22_O6	Serum	Duzhong
CF20	Cryptotanshinone	35825–57-1	C_19_H_20_O_3_	Serum	Danshen
CF21	Pinoresinol diglucoside	63902–38-5	C_32_H_42_O_16_	Serum, Brain	Duzhong
CF22	Pinoresinol glucoside	41607–20-9	C_26_H_32_O_11_	Serum	Duzhong

### The PPI network and functional enrichment of potential targets

A total of 55 intersection targets were screened out and used to construct the PPI network., Subsequently, a thorough topology analysis was executed employing a Network Analyzer. The magnitude and intensity of each node were indicative of its degree value, with greater size and deeper color denoting higher degree values. We noted that six nodes, including IL-6, HIF-1α, VEGF, IL-1β, TNF and STAT3, exhibited higher degrees, indicating their potential role as hub proteins within the network (Fig. 3C).

**Fig. 4:**
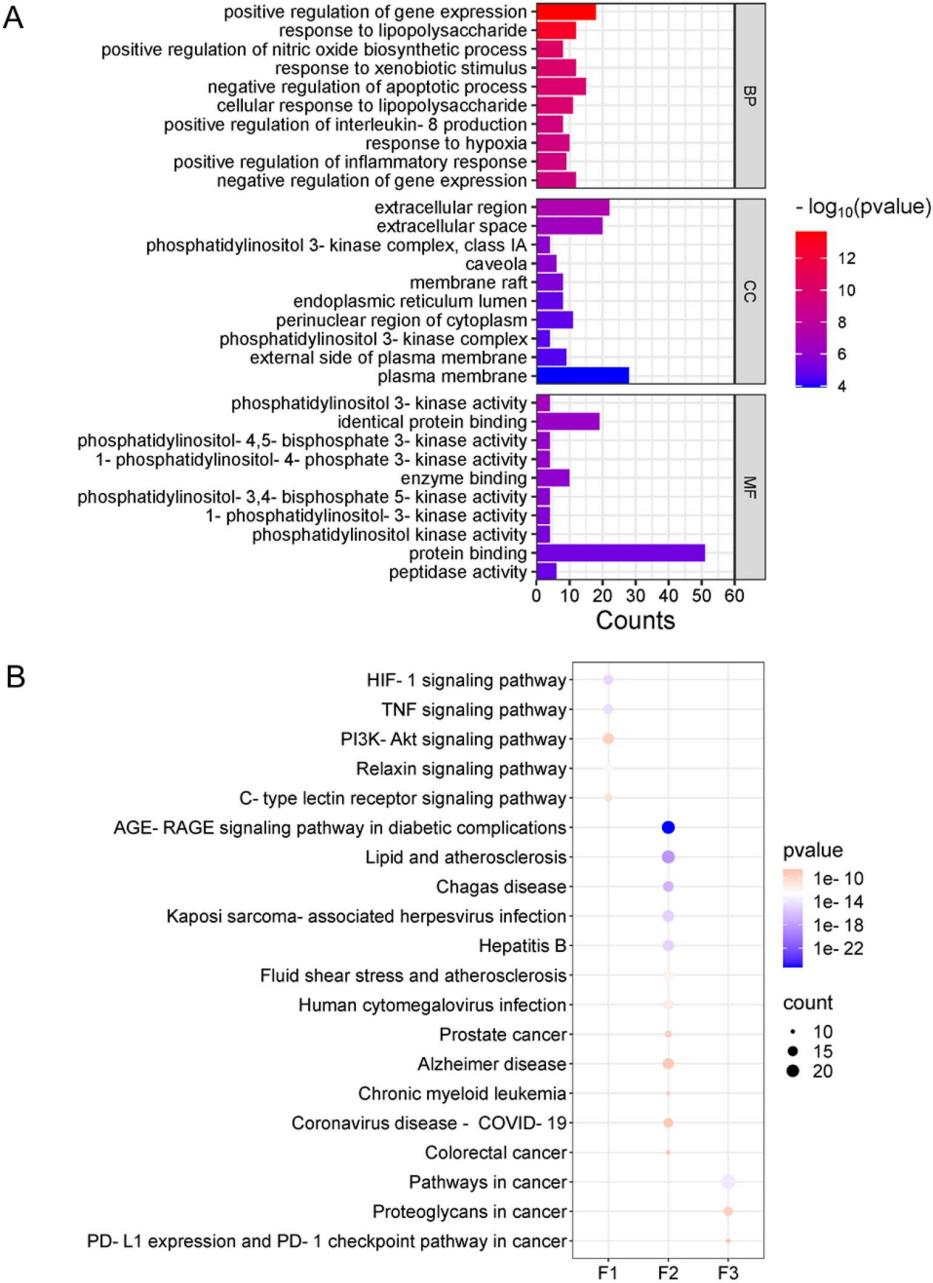
The analysis of functional enrichment in potential targets. **A** GO enrichment analysis by the DAVID database (https://david.ncifcrf.gov/home.jsp). **B** The top 20 enriched KEGG pathways of SMJF Granule candidate targets

To elucidate the molecular mechanisms underlying SMJF Granule in CI, we conducted GO and KEGG enrichment analyses on a dataset of 55 candidate targets. The GO analysis (Fig. 4A) yielded a comprehensive list of 436 biological processes, 42 cellular components, and 67 molecular functions. The biological processes mainly involve pathways such as response to hypoxia, positive regulation of nitric oxide biosynthetic process, positive regulation of inflammatory response, and positive regulation of interleukin-8 production, which are highly likely to be closely associated with the HIF-1 pathway.

**Fig. 5:**
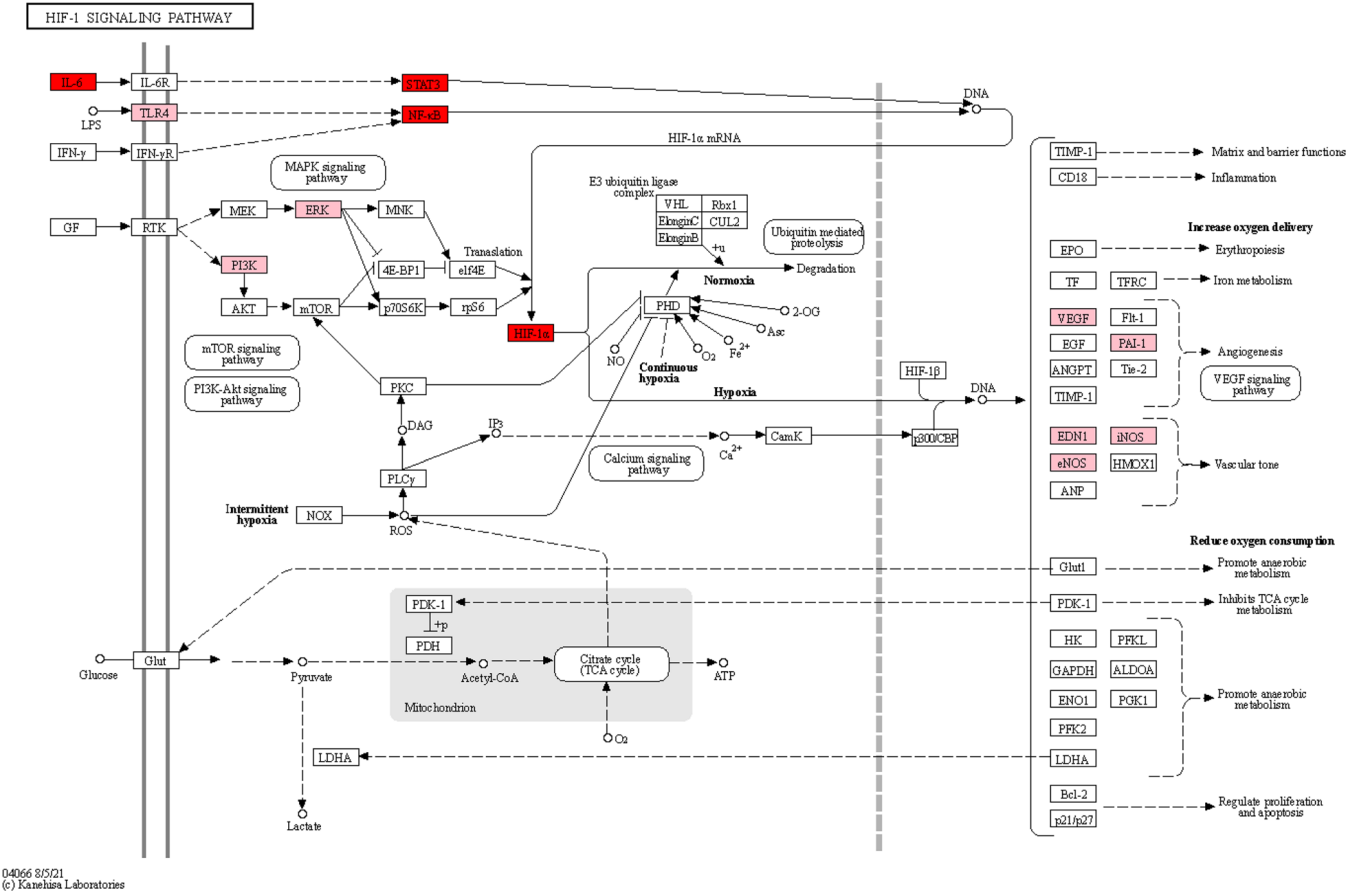
The HIF-1 signaling pathway. Data was retrieved from the KEGG database (https://www.genome.jp/kegg/). The red rectangles represent the core targets and the pink rectangles represent the candidate targets of SMJF Granule in the treatment of CI

Furthermore, KEGG enrichment analysis revealed 151 pathways. The top 20 terms were selected based on their significance (p < 0.05). A visual representation of these enriched pathways, displayed in a dot plot (Fig. 4B), demonstrated that a majority of these pathways were enriched with multiple targets associated with cerebral ischemia. In particular, it was evident that several proteins, such as HIF-1α, IL-6, and VEGF, played integral roles within the HIF-1 pathway (Fig. 5).

### Identification of differentially expressed genes in rat brain tissues

**Fig. 6:**
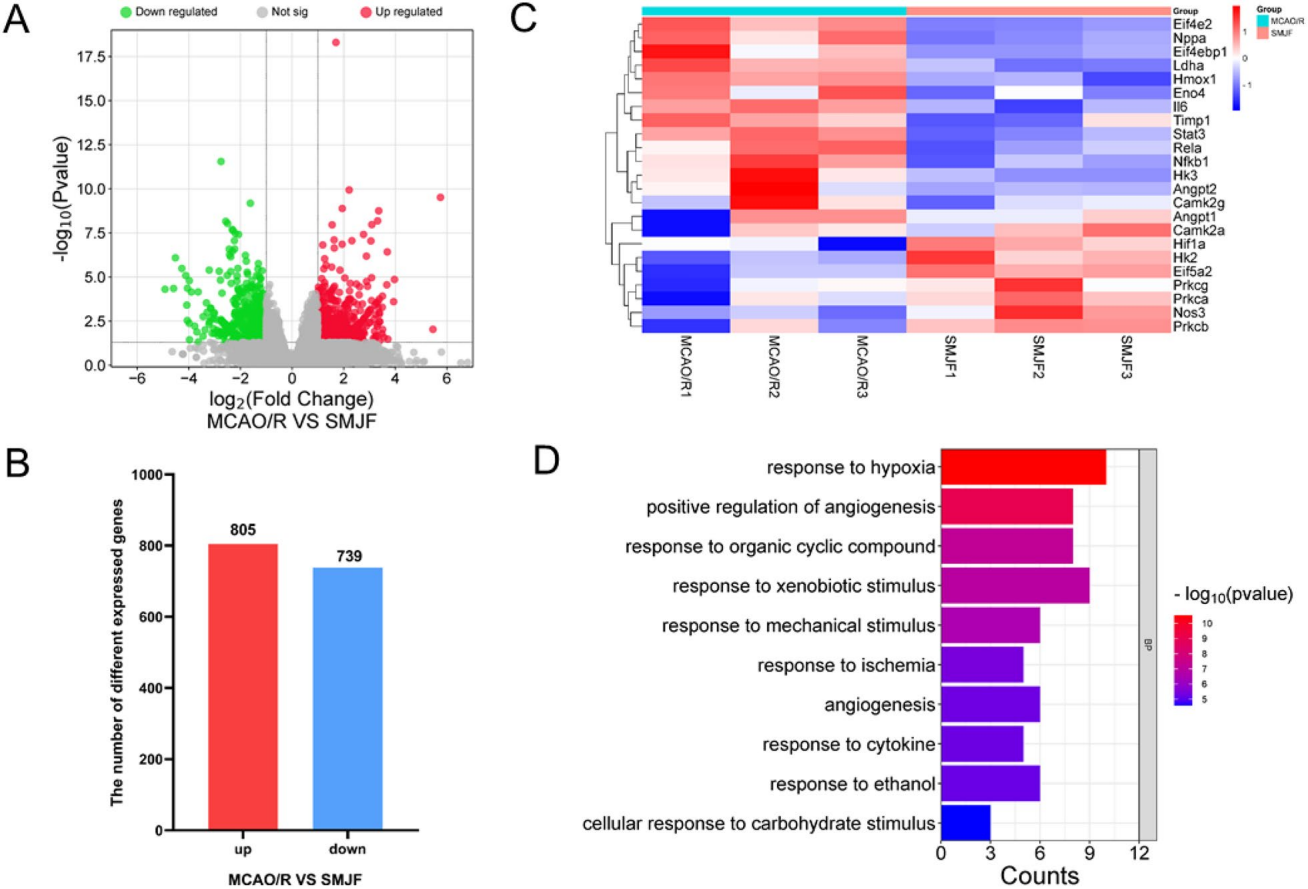
Transcriptomic analysis results of MCAO/R rats treated with SMJF Granule. **A** Volcano plots of DEGs in MCAO/R and SMJF group. **B** Identification of DEGs (p-value < 0.05, |log2FC|≥ 1). **C** Heatmap of relevant DEGs in the HIF1-α signaling pathway. **D** The top ten biological processes of the enrichment analysis in HIF1-α signaling pathway related DEGs

As shown in Fig. 6, significant variations were observed in transcripts between the MCAO/R group and the SMJF group. In this way, 1544 DEGs were screened in the SMJF group including 805 upregulated and 739 downregulated compared to the MCAO/R group (Fig. 6A, B). The results of KEGG pathway enrichment analysis primarily involve HIF-1 signaling pathway, TNF signaling pathway, NF-κB signaling pathway and so on. The biological processes mainly involve inflammatory response, positive regulation of angiogenesis and so on. Therefore, both network pharmacology and transcriptomics analysis indicated that the therapeutic effect of SMJF on CI was highly correlated with the HIF-1 signaling pathway. To better derive and verify the previous analysis, we conducted heatmap and enrichment analysis on DEGs involving the HIF-1 signaling pathway (Fig. 6C, D). The results revealed that SMJF Granule could regulate the expression of HIF1-α related genes, thereby influencing several biological processes such as response to hypoxia, positive regulation of angiogenesis, and so on.

### Analyzing molecular docking and SPR affinity assessment

**Fig. 7:**
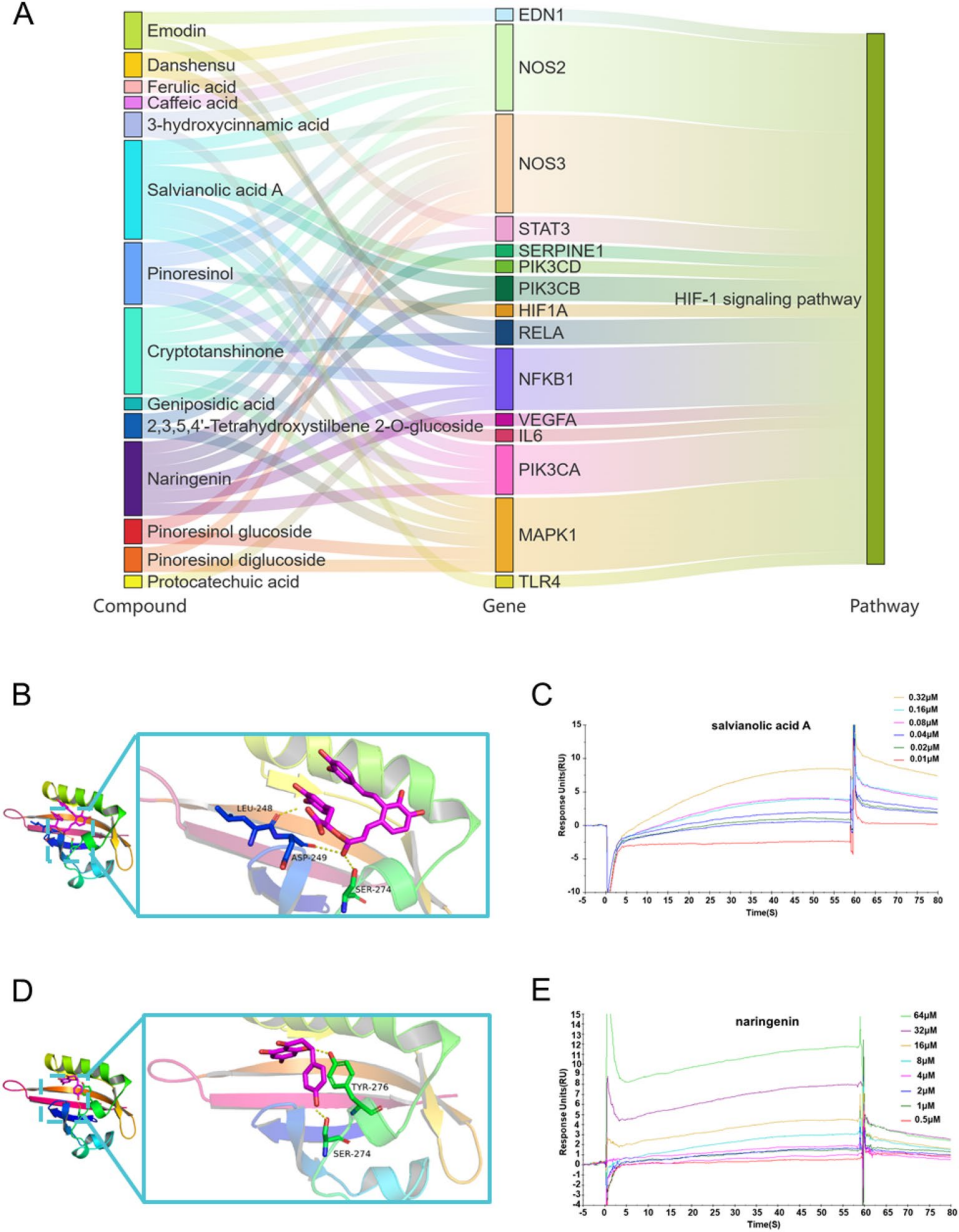
The docking and SPR analysis of key compounds and HIF-1α protein. **A** Alluvial map of the main active ingredients of SMJF Granule in ischemic stroke related to the HIF-1 signaling pathway. The alluvial plot was visualized using the online OmicShare tools (https://www.omicshare.com/tools). **B**, **C** The docking pose and SPR assay of salvianolic acid A binding to HIF-1α protein. **D**, **E** The docking pose and SPR assay of naringenin binding to HIF-1α protein

Based on the outcomes of network pharmacology and the alluvial plot analysis (Fig. 7A) about the HIF-1 pathway, we analyzed the top ten compounds that were identified as having the highest values in the herbs-ingredients-targets network. Among them, the docking score for salvianolic acid A (Fig. 7B) and the HIF-1α protein was determined to be − 6.3 kcal/mol, coincidentally, the same applies to naringenin (Fig. 7D).

To further validate the interaction between salvianolic acid A and naringenin with the HIF-1α protein, we conducted a SPR-based binding assay to determine the affinity constants. The results revealed that salvianolic acid A (Fig. 7C) and naringenin (Fig. 7E) displayed a direct binding affinity to the HIF-1α protein at the micromolar level, with corresponding KD values of 0.115 μM and 92.29 μM. These results suggested a substantial interaction between these compounds and the HIF-1α protein, potentially contributing to their role in the treatment of cerebral ischemia.

### SMJF granule attenuated ischemia–reperfusion injury

**Fig. 8:**
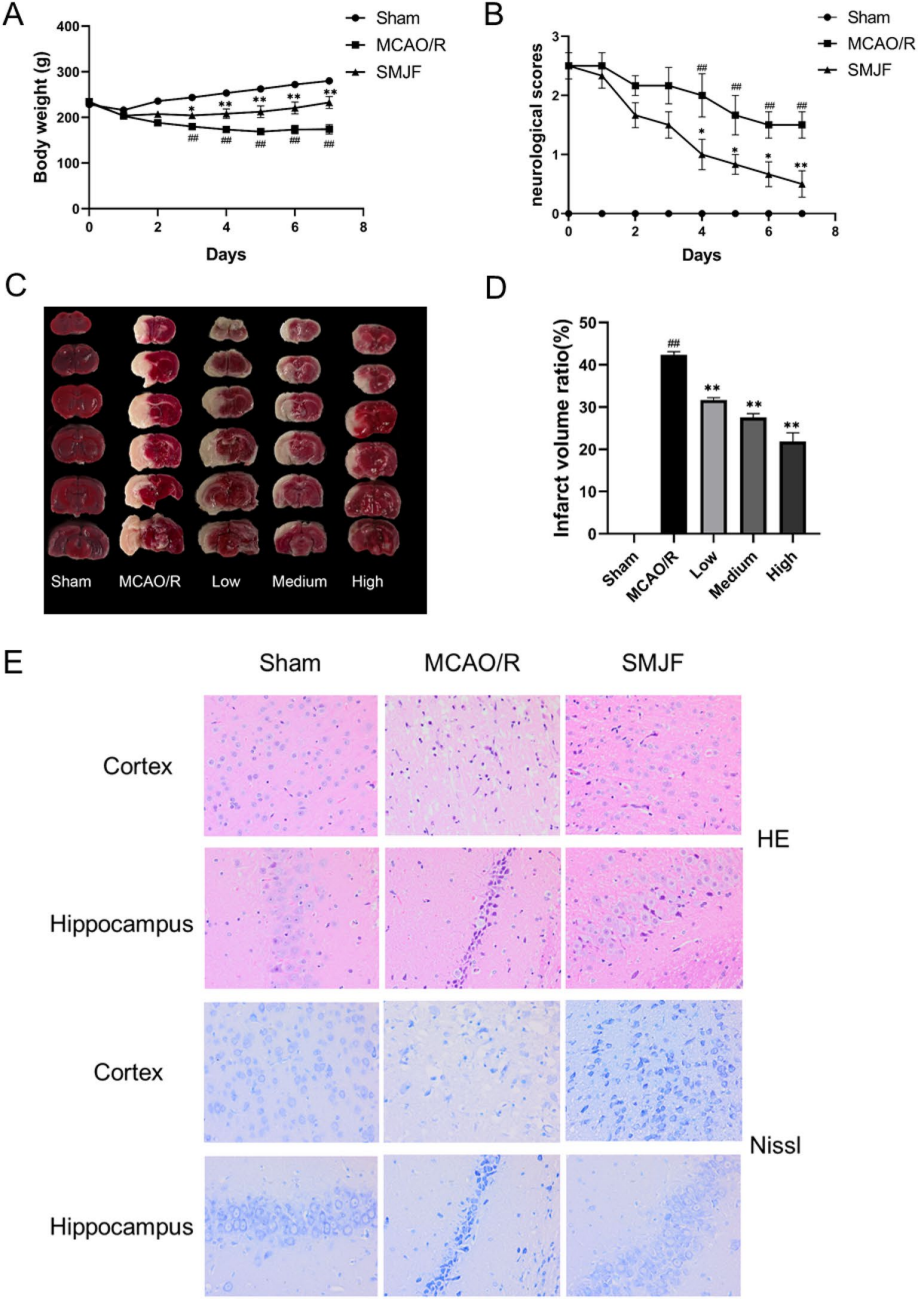
SMJF Granule treatment alleviates brain damage induced by experimental stroke. **A** Body weight was assessed daily for seven consecutive days post-surgery. **B** Neurological impairment scores of MCAO/R rats. **C** Representative images of TTC-stained sections. **D** Quantitative evaluation of infarct volume. **E** Typical micrographs of HE and Nissl staining of cortex and hippocampus. All data were presented as mean ± SEM. ^#^*p* < 0.05, ^##^*p* < 0.01 versus sham group; *p < 0.05, **p < 0.01, versus model group, respectively

To confirm our previous findings, animal models were established, including sham, MCAO/R, and SMJF Granule groups. Compared to the sham control group, MCAO/R modeling rats exhibited a significant weight loss, which could be reversed by SMJF Granule treatment (Fig. 8A). After the operation, ischemic rats showed a gradual improvement in neurological deficits, suggesting mild recovery post-MCAO. SMJF Granule treatment significantly reduced neurological deficit scores at 4, 5, 6, and 7 days compared to the MCAO/R group (Fig. 8B). Consistent with earlier findings, transient MCAO surgery significantly elevated infarct sizes in the experimental animals. Fortunately, the administration of varying doses (0.514 g/kg, 1.2 g/kg, 3.43 g/kg) of SMJF Granule effectively reduced the infarction volumes induced by stroke (Fig. 8C, D).

As depicted in Fig. 8E, ischemic alterations were observed in the cerebral cortex and hippocampus of the MCAO/R group, characterized by the reduction of healthy neurons, obvious nerve cell loss, aberrant cell shape, and cellular degeneration. SMJF Granule exhibited a superior ability to facilitate this phenomenon. Nissl staining in the sham group displayed well-preserved neurons in the cortex and hippocampus with orderly arrangement and healthy cell structures. In contrast, the MCAO/R group exhibited damaged neurons with reduced density, disordered arrangement, and cell shrinkage. Compared to the model group, the SMJF group demonstrated improvement in pathological brain tissue damage, with cell morphology approaching normalcy. These data showed that SMJF Granule might exert an effect on improving the ischemia–reperfusion injury in MCAO/R rats.

### The administration of SMJF granule partially ameliorated the mRNA and protein expressions of the candidate targets identified

**Fig. 9:**
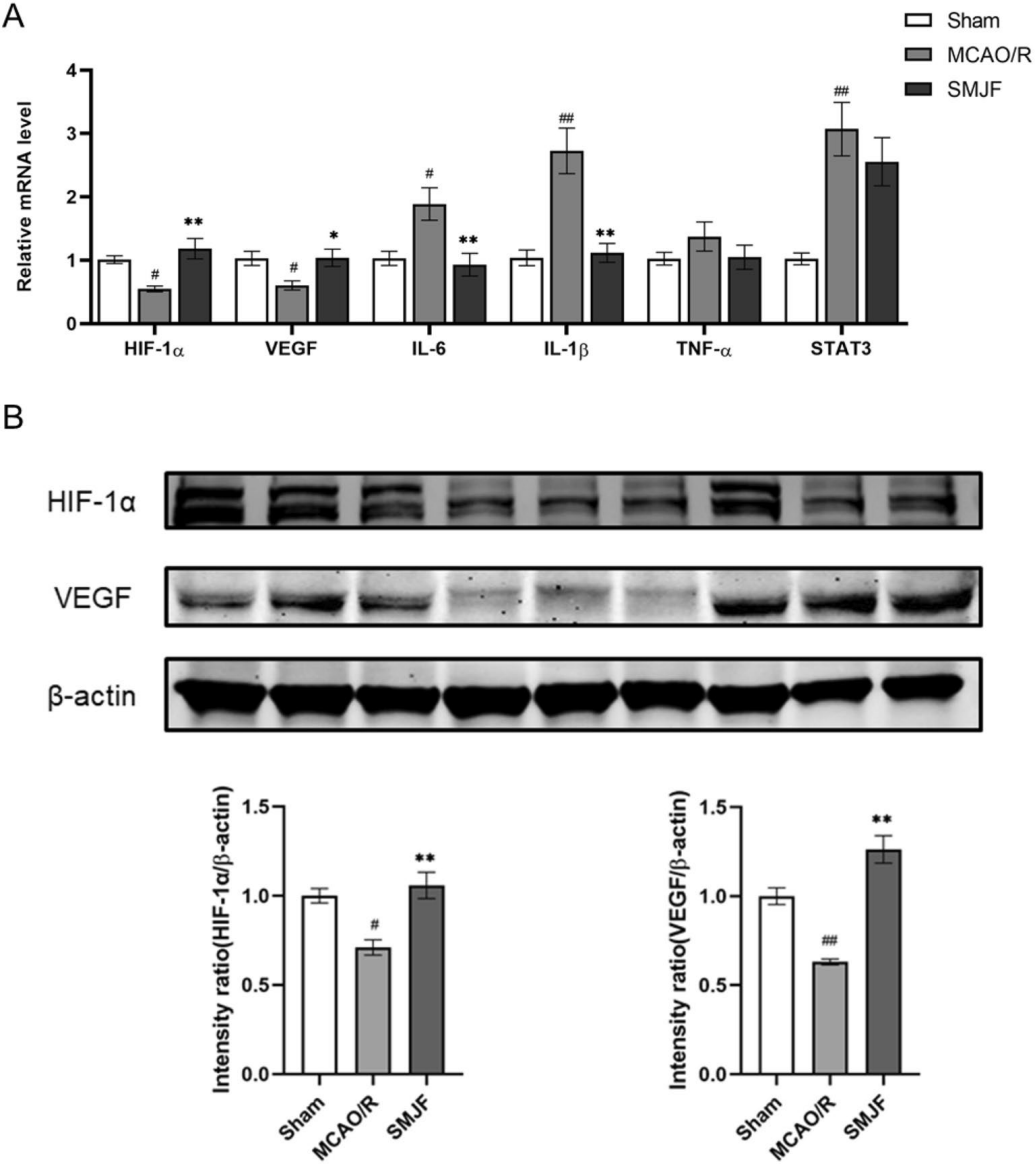
The SMJF Granule exhibited partial restoration of mRNA and protein levels in the candidate targets. **A** RT-qPCR analysis was conducted to determine the relative mRNA expression of key targets. **B** Western-blot analysis was performed to assess the protein expressions. Data are presented as mean ± SEM; ^#^*p* < 0.05, ^##^*p* < 0.01 vs sham group; *p < 0.05, **p < 0.01vs model group

To verify the key targets of SMJF Granule in MCAO/R therapy, the mRNA and protein expression levels of these targets were assessed by RT-qPCR and western blotting. The results showed that the mRNA levels of HIF-1α, VEGF, IL-6, and IL-1β were reversed after SMJF treatment (Fig. 9A). Consistent with this, the protein levels of HIF-1α and VEGF were significantly increased in SMJF group (Fig. 9B).

### SMJF granule strengthened angiogenesis in MCAO/R rats

**Fig. 10:**
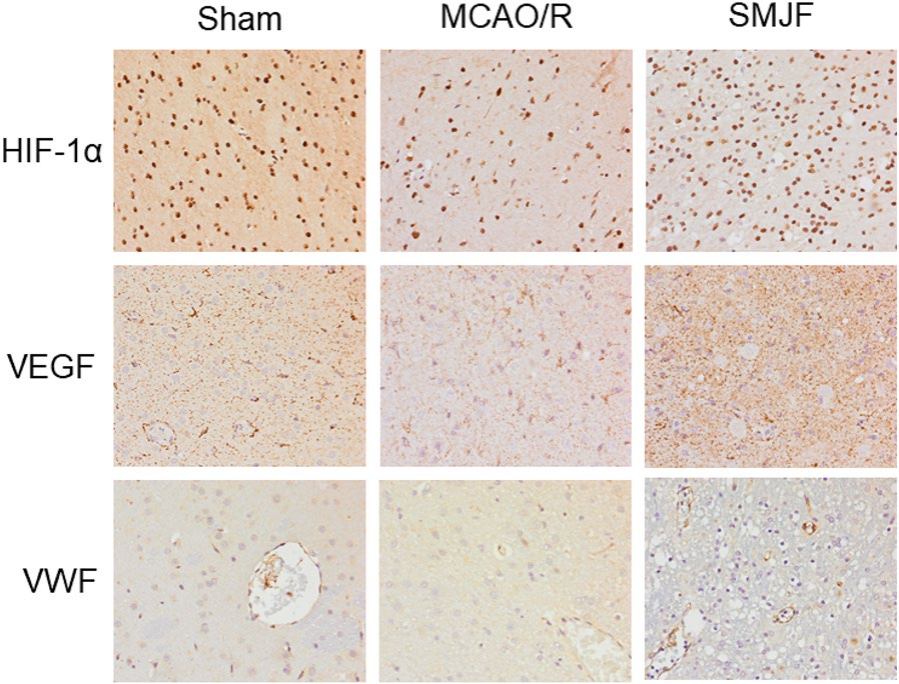
Representative images showed IHC staining of HIF-1α, VEGF, and vWF in the cerebral cortex (magnification 400 ×)

Immunohistochemical experiments revealed that the HIF-1α-positive cell density of the SMJF group significantly increased compared to the MCAO/R model group (Fig. 10). Furthermore, the SMJF Granule treatment also led to an increase in VEGF and vWF expression in the cerebral cortex. vWF in the cytoplasm of endothelial cells is labeled as a brownish-yellow particle, and any endothelial cells or clusters stained brown are usually considered as vessel count. Taken together, these results indicated that SMJF Granule was able to promote angiogenesis in the lesion area of the brain in MCAO/R rats.

## Discussion

Post-stroke neurologic and behavioral deficits have emerged as a significant health concern, severely impacting the quality of life in stroke survivors [14]. Angiogenesis and neurogenesis are considered crucial neurovascular responses in stroke recovery [15]. Increased microvessel density has been observed in the peri-infarct regions of ischemic stroke patients [16]. At least one study suggests that the quantity of newly formed blood vessels appears to be associated with longer survival in ischemic stroke patients, implying the potential benefits of active angiogenesis [17]. Therefore, delving into drugs targeting angiogenesis represents a meaningful avenue for treating cerebral ischemic injuries. For instance, Fluoxetine can upregulate protein expression in the HIF-1α-Netrin/VEGF cascade, promoting angiogenesis and improving long-term functional recovery after ischemic stroke [18].

The SMJF Granule holds promise as a prospective therapeutic agent for ischemic stroke recovery. In the previous research, the monarch drugs in this prescription such as danshen may reduce the infarct area in rat brains suffering from ischemia–reperfusion injury by effectively scavenging free radicals [19]. Additionally, *Angelica sinensis* activates the p38MAPK/HIF-1α/VEGF signaling pathway to promote angiogenic and anti-apoptotic effects against cerebral ischemia–reperfusion injury in rats [20]. Moreover, Chuanxiong shows potential as a prospective therapeutic agent for treating neuronal damage following cerebral ischemia due to its anti-neuroinflammatory properties [21]. However, the specific active ingredients responsible for this effect have not been definitively identified and confirmed. Therefore, we conducted an integrated approach involving network pharmacology and transcriptomics to clarify the potential mechanisms of SMJF Granule. The results revealed that SMJF Granule primarily exerted its effects in promoting angiogenesis, anti-inflammation, and anti-apoptosis activities, particularly in connection with the HIF-1 pathway. Furthermore, our observations suggested that SMJF Granule might ameliorate cerebral ischemia–reperfusion injury by modulating key targets such as HIF-1α, IL-6, and NF-κB1, which are enriched in the HIF-1 signaling pathway.

Hypoxia-inducible factor 1 (HIF-1) is composed of two subunits, HIF-1α and HIF-1β. HIF-1α is considered a crucial maintenance of oxygen homeostasis and is tightly controlled by by the levels of oxygen [22]. It plays a wide-ranging role in the pathophysiology of ischemic stroke, including inflammation, angiogenesis, neuronal apoptosis, and glucose metabolism [23]. Previous research has revealed that the expression of HIF-1α is not limited to neurons but is also observed in other cell types such as astrocytes, endothelial cells, and microglia, displaying cell-type-specific roles [24]. Under conditions of low oxygen, HIF-1α governs internalized adaptive mechanisms by controlling multiple signaling pathways and downstream genes [25]. Upon initiation, HIF-1α stimulates gene transcription linked with adjustment and resilience. Convincing evidence indicates that after cerebral ischemic injury, VEGF is strongly induced through the HIF-1α pathway [26]. VEGF, a highly potent cytokine that enhances endothelial growth, triggers the proliferation of endothelial cells and actively contributes to the process of angiogenesis and the formation of new blood vessels in ischemic injury tissues [27]. Given the pivotal role of HIF-1α in ischemic and hypoxic injuries, drug development targeting HIF-1α has become a research hotspot. The HIF-1α activator Roxadustat (FG-4592), a HIF hydroxylase inhibitor, is currently being investigated for anemia treatment [28]. Recently, there have been reports suggesting that FG-4592 exhibits notable neuroprotective effects, indicating potential in the treatment of Parkinson's disease [29, 30]. In this study, molecular docking and SPR analyses revealed promising interactions between HIF-1α protein and compounds such as naringenin and salvianolic acid A.

Naringenin has been recognized as a promising neuroprotective agent, exerting its anti-inflammatory, anti-apoptotic, and antioxidant mechanisms through the inhibition of the NF-κB signaling pathway. Simultaneously, naringenin positively affects brain I/R injury by regulating the HIF-1α/AKT/mTOR signaling pathway [31–33]. Salvianolic acid A has been shown to alleviate cerebral ischemia–reperfusion injury by inhibiting inflammation and apoptosis, promoting neurogenesis, and suppressing MMP-9 expression [34, 35]. The angiogenesis-promoting effect observed in SMJF Granule may be related to these key active constituents. However, it is worth noting that there is currently a lack of experimental research to confirm the impact of salvianolic acid A on HIF-1α. Therefore, further investigation of this potential interaction is warranted.

This study lies in the utilization of network pharmacology and transcriptomics to predict and preliminarily validate the active ingredients, biological processes, and mechanisms of SMJF Granule against cerebral ischemia. Our research indicates that SMJF Granule may exert its anti-CI effects by modulating the expression of HIF-1α and VEGF, thereby promoting angiogenesis. The advantages of TCM formulations, with their multi-ingredient and multi-target regulation, offer promising prospects for alleviating ischemic injuries.

## Conclusion

By leveraging UPLC-Q-TOF-MS, network pharmacology, transcriptomics, and experimental verification methods, we have identified the active ingredients and potential targets associated with SMJF Granule in the treatment of cerebral ischemia. Our research results indicate that SMJF Granule can modulate the expression of HIF-1α and VEGF, thereby conferring angiogenesis against ischemic injuries.

### Supplementary Information

**Additional file 1: Table S1. **Identified ingredients of SMJF Granule.

## Declarations

### Data availability

The raw data of the original study will be made available by the authores. Further inquiries can be directed to the corresponding authors.
