# Supplementary material for: Compound Shenma Jingfu granule alleviates cerebral ischemia via HIF-1α-mediated promotion of angiogenesis
Source: Chin Med. 2024 Apr 10;19:62. doi: 10.1186/s13020-024-00926-w (PMC11005288; doi:10.1186/s13020-024-00926-w)
Supplement: Supplementary file 1 — Additional file 1: Table S1. Identified ingredients of SMJF Granule. [file 13020_2024_926_MOESM1_ESM.docx]

**Table S1.** Identified ingredients of SMJF Granule

| No. | Time (min) | Adduct ion | *M/Z* | PPM | Formula | Molecular weight | Identification | MS/MS |
| --- | --- | --- | --- | --- | --- | --- | --- | --- |
| 1 | 2.06 | [M+FA-H]^-^ | 331.1035 | 0.1 | C_13_H_18_O_7_ | 286.11 | Gastrodin | 331.0661;271.0432;169.0152;123.0454 |
| 2 | 2.2 | [M-H]^-^ | 169.0151 | 5 | C_7_H_6_O_5_ | 170.02 | Gallic acid | 169.0137;125.0234;107.0123 |
| 3 | 3.07 | [M-H]^-^ | 257.1152 | 3.5 | C_11_H_18_N_2_O_5_ | 258.12 | gamma-L-Glutamyl-L-pipecolic acid | 213.1272;195.1122;128.0354 |
| 4 | 3.69 | [M-H]^-^ | 197.046 | 2.3 | C_9_H_10_O_5_ | 198.05 | Danshensu | 197.0436;179.0358;162.8392;135.0454;123.0455 |
| 5 | 3.91 | [M-H]^-^ | 391.1254 | 2.1 | C_16_H_24_O_11_ | 392.13 | Shanzhiside | 391.1241;229.0711;211.0607;185.0812;167.0717 |
| 6 | 4.51 | [M-H]^-^ | 373.116 | 5.3 | C_16_H_22_O_10_ | 374.12 | Geniposidic acid | 373.1165;211.0604;149.0606;123.0448 |
| 7 | 4.56 | [M-H]^-^ | 153.0206 | 8.3 | C_7_H_6_O_4_ | 154.03 | Protocatechuic acid | 153.0216;123.0492;109.0293;108.0222 |
| 8 | 5.21 | [M+FA-H]^-^ | 449.1308 | 1.6 | C_17_H_24_O_11_ | 404.13 | Feretoside | 403.1183;241.0709 |
| 9 | 6.61 | [M-H]^-^ | 353.0873 | -1.4 | C_16_H_18_O_9_ | 354.10 | Neochlorogenic acid | / |
| 10 | 6.84 | [M-H]^-^ | 345.1559 | 1.2 | C_16_H_26_O_8_ | 346.16 | Jasminoside B | 179.0550;165.0927;121.1026 |
| 11 | 7.37 | [M-H]^-^ | 137.0245 | 0.6 | C_7_H_6_O_3_ | 138.03 | Protocatechualdehyde | 137.0243;108.0225 |
| 12 | 8.45 | [M+FA-H]^-^ | 449.1323 | -2.5 | C_17_H_24_O_11_ | 404.13 | Gardenoside | 403.1224;241.0703;139.0398 |
| 13 | 9.06 | [M+H]^+^ | 247.1441 | 0 | C_14_H_18_N_2_O_2_ | 246.14 | Cyclo(Val-Phe) | 188.070;146.0594;118.0642 |
| 14 | 9.22 | [M+FA-H]^-^ | 449.131 | 2.1 | C_17_H_24_O_11_ | 404.13 | Deacetyl asperulosidic acid methyl ester | 449.1306;241.0683;127.0395 |
| 15 | 9.49 | [M-H]^-^ | 459.1151 | 1.5 | C_19_H_24_O_13_ | 460.12 | Parishin E | 459.1150;173.0084;129.0191;111.0084 |
| 16 | 9.5 | [M-H]^-^ | 375.1291 | -1.5 | C_16_H_24_O_10_ | 376.14 | Loganic acid | 375.1299;213.0783;169.0884;151.0764 |
| 17 | 9.71 | [M+H]^+^ | 414.131 | 3.5 | C_17_H_23_N_3_O_7_S | 413.13 | S-( 4-hydroxybenzyl) -giutathione | 414.1386;339.0986;308.0881;233.0596;179.0486;162.0215 |
| 18 | 9.87 | [M+FA-H]^-^ | 451.1481 | 5.3 | C_17_H_26_O_11_ | 406.15 | Shanzhiside methyl ester/Morroniside | 451.1571;405.1390;243.0866;179.0556;155.0343;141.0548 |
| 19 | 10.05 | [M-H]^-^ | 123.0452 | 0 | C_7_H_8_O_2_ | 124.05 | p-Hydroxybenzyl alcohol | / |
| 20 | 10.39 | [M-H]^-^ | 353.0859 | 1.7 | C_16_H_18_O_9_ | 354.10 | Chlorogenic acid | 191.0548;173.0435;161.0222;135.0440 |
| 21 | 10.79 | [M+FA-H]^-^ | 595.1855 | -4.2 | C_23_H_34_O_15_ | 550.19 | Genipin 1-gentiobioside | 595.1949;549.1846;225.0775;207.0669;123.0458 |
| 22 | 10.88 | [M-H]^-^ | 353.0873 | -1.4 | C_16_H_18_O_9_ | 354.10 | Cryptochlorogenic acid | 353.0860;191.0561;179.0347;173.0452;135.0455 |
| 23 | 11.75 | [M+FA-H]^-^ | 433.1362 | 2.4 | C_17_H_24_O_10_ | 388.14 | Geniposide | 433.1374;387.1281;225.0767;207.0656;123.0454 |
| 24 | 12.03 | [M+FA-H]^-^ | 403.125 | 1 | C_16_H_22_O_9_ | 358.13 | Sweroside | 403.1344;357.1187;195.0649;125.0245 |
| 25 | 12.12 | [M+FA-H]^-^ | 435.1519 | 2.5 | C_17_H_26_O_10_ | 390.15 | Loganin | 435.1531;227.0920;127.0395 |
| 26 | 12.51 | [M-H]^-^ | 593.1539 | 4.6 | C_27_H_30_O_15_ | 594.16 | Vicenin-2 | 593.1537;503.1241;473.1101;383.0788;353.0678 |
| 27 | 12.59 | [M-H]^-^ | 727.2078 | -1.8 | C_32_H_40_O_19_ | 728.22 | Parishin B | 727.2096;441.1051;423.0938;161.0459 |
| 28 | 13.09 | [M-H]^-^ | 505.1576 | 2.6 | C_21_H_30_O_14_ | 506.16 | Logmalicid A | 505.1533;487.1443;227.0913;127.0397;115.0037 |
| 29 | 13.17 | [M-H]^-^ | 681.2405 | 0.7 | C_32_H_42_O_16_ | 682.25 | Pinoresinol Diglucoside | 405.1224;357.1350;161.0477;151.0410 |
| 30 | 13.23 | [M-H]^-^ | 727.2094 | 0.4 | C_32_H_40_O_19_ | 728.22 | Parishin C | 727.2043;681.2454;519.1948;357.1359;161.0476;151.0405 |
| 31 | 13.63 | [M-H]^-^ | 505.1593 | 6 | C_21_H_30_O_14_ | 506.16 | Logmalicid B | 505.1543;487.1435;227.0912;209.0810;127.0393 |
| 32 | 14.28 | [M-H]^-^ | 359.1345 | -0.7 | C_16_H_24_O_9_ | 360.14 | Deoxyloganic acid | / |
| 33 | 14.7 | [M-H]^-^ | 193.0511 | 2.4 | C_10_H_10_O_4_ | 194.06 | Ferulic acid | 193.0499;179.0301;134.0379 |
| 34 | 15.19 | [M+NH_4_]^+^ | 1014.3488 | 3.9 | C_45_H_56_O_25_ | 996.31 | Parishin A | 1014.3500;535.1781;299.0758;213.0912;107.0501 |
| 35 | 15.56 | [M-H]^-^ | 405.1184 | -1.7 | C_20_H_22_O_9_ | 406.13 | 2,3,5,4'-Tetrahydroxystilbene 2-O-glucoside | 405.1183;243.0662;225.0559;173.0616;149.0247;137.0235 |
| 36 | 16.73 | [M+H]^+^ | 581.1886 | 3.6 | C_27_H_32_O_14_ | 580.18 | Naringin | 419.1391;383.1151;339.0858;273.0775;263.0557;153.0196 |
| 37 | 16.76 | [M+FA-H]^-^ | 793.2784 | 1.5 | C_33_H_48_O_19_ | 748.28 | Sylvestroside I | 747.2702;585.2065;485.1596;375.1232;227.0925 |
| 38 | 16.78 | [M-H]^-^ | 515.1203 | 1.6 | C_25_H_24_O_12_ | 516.13 | Isochlorogenic acid B | 515.1181;353.0855;203.0322;191.0535;179.0366;173.0477 |
| 39 | 16.97 | [M-H]^-^ | 537.1042 | 0.7 | C_27_H_22_O_12_ | 538.11 | Salvianolic acid I | 537.1048;339.0504;295.0610;239.0723 |
| 40 | 17.27 | [M+FA-H]^-^ | 475.1491 | 7.1 | C_19_H_26_O_11_ | 430.15 | 10-O-acetylgeniposide | 475.1512;311.0562;269.0445;267.0902;207.0669 |
| 41 | 17.3 | [M-H]^-^ | 515.1191 | -0.8 | C_25_H_24_O_12_ | 516.13 | Isochlorogenic acid A | 515.1134;353.0886;191.0568;179.0357 |
| 42 | 17.59 | [M-H]^-^ | 187.0983 | 3.8 | C_9_H_16_O_4_ | 188.10 | Eucommiol | 187.0982;125.0982;97.0659 |
| 43 | 17.64 | [M+H]^+^ | 300.1236 | 1.9 | C_17_H_17_NO_4_ | 299.12 | N-trans-caffeoyltyramine | 300.1215;163.0367;145.0254;121.0634 |
| 44 | 17.71 | [M-H]^-^ | 541.1572 | 1.7 | C_24_H_30_O_14_ | 542.16 | Cornuside | 541.1549;347.0775;189.0582;169.0136 |
| 45 | 17.9 | [M-H]^-^ | 609.1863 | 6.2 | C_28_H_34_O_15_ | 610.19 | Hesperidin | 609.1807;301.0705;286.0475 |
| 46 | 18.17 | [M-H]^-^ | 717.1451 | -1.4 | C_36_H_30_O_16_ | 718.15 | Salvianolic acid E | 717.1499;519.0948;339.0495;321.0399 |
| 47 | 18.19 | [M+H]^+^ | 207.1017 | 0.6 | C_12_H_14_O_3_ | 206.09 | Senkyunolide F | 207.1053;189.0925;179.1068;165.0545;133.1023;117.0697;91.0528 |
| 48 | 18.29 | [M-H]^-^ | 515.1227 | 6.2 | C_25_H_24_O_12_ | 516.13 | Isochlorogenic acid C | 515.1197;353.0865;191.0560;179.0331;173.0445 |
| 49 | 18.4 | [M-H]^-^ | 695.2187 | -0.8 | C_32_H_40_O_17_ | 696.23 | 6''-O-trans-Coumaroyl genipin gentiobioside | 695.2218;469.1333;265.0728;225.0768 |
| 50 | 18.51 | [M-H]^-^ | 359.0782 | 2.7 | C_18_H_16_O_8_ | 360.08 | Rosmarinic acid | 359.0735;197.0448;179.0342;161.0242 |
| 51 | 18.66 | [M-H]^-^ | 755.2439 | 4.6 | C_34_H_44_O_19_ | 756.25 | 6″-O-[trans-Sinapoyl] genipin gentiobioside | 755.2400;529.1570;427.1303;265.0745 |
| 52 | 18.82 | [M-H]^-^ | 725.2292 | -0.9 | C_33_H_42_O_18_ | 726.24 | 6″-O-[trans-Feruloyl] genipin gentiobioside | 725.2251;499.1448;301.0740;207.0674;193.0512 |
| 53 | 19.02 | [M-H]^-^ | 537.1045 | 1.2 | C_27_H_22_O_12_ | 538.11 | Salvianolic acid H | 493.1147;313.0705;295.0608;185.0243 |
| 54 | 19.2 | [M-H]^-^ | 431.0973 | -2.5 | C_21_H_20_O_10_ | 432.11 | Emodin-1-O-β-D-glucopyranoside | 431.0959;269.0437;240.0413 |
| 55 | 19.21 | [M+H]^+^ | 207.1013 | -1.3 | C_12_H_14_O_3_ | 206.09 | Senkyunolide F isomer | 189.0885;161.0926;143.0831;123.0443;91.0532 |
| 56 | 20.03 | [M+H]^+^ | 284.128 | -0.4 | C_17_H_17_NO_3_ | 283.12 | p-coumaroyltyramine | 147.0436;121.0645;119.0486;91.0543 |
| 57 | 20.24 | [M-H]^-^ | 717.1455 | -0.8 | C_36_H_30_O_16_ | 718.15 | Salvianolic acid B | 717.1460;519.0920;33.0478;321.0387 |
| 58 | 20.41 | [M+FA-H]^-^ | 1519.516 | 1.1 | C_66_H_90_O_37_ | 1474.52 | Dipsanoside A | / |
| 59 | 20.59 | [M+H]^+^ | 328.1175 | -1.4 | C_18_H_17_NO_5_ | 327.11 | Terrestriamide | 177.0543;145.0277;117.0325 |
| 60 | 20.78 | [M+H]^+^ | 314.137 | -5.4 | C_18_H_19_NO_4_ | 313.13 | N-trans-feruloyltyramine | 177.0536;145.0280;121.0653;117.0341 |
| 61 | 21.48 | [M-H]^-^ | 717.1467 | 0.8 | C_36_H_30_O_16_ | 718.15 | Salvianolic acid Y | 717.1478;519.0934;339.0514;321.0397 |
| 62 | 21.58 | [M+H]^+^ | 441.2013 | -1.6 | C_24_H_28_N_2_O_6_ | 440.19 | Terrestribisamide | 265.1530;248.1288;177.0566;145.0284 |
| 63 | 21.84 | [M-H]^-^ | 493.1165 | 5 | C_26_H_22_O_10_ | 494.12 | Salvianolic acid A | 493.1109;313.0813;295.0594;203.0360 |
| 64 | 22.08 | [M-H]^-^ | 593.1889 | 2.2 | C_28_H_34_O_14_ | 594.19 | Poncirin | 593.1952;309.0720;285.0735 |
| 65 | 22.43 | [M-H]^-^ | 407.1353 | 1.3 | C_20_H_24_O_9_ | 408.14 | Torachrysone 8-glucoside | 407.1292;245.0811;230.0576;215.0332 |
| 66 | 22.84 | [M-H]^-^ | 431.2844 | 2.6 | C_21_H_20_O_10_ | 432.11 | Emodin-8-β-D-glucoside | 431.0976;311.0566;269.0436;225.0545 |
| 67 | 23.88 | [M+H]^+^ | 728.3986 | 1.2 | C_36_H_53_N_7_O_9_ | 727.39 | Citrusin Ⅲ | 728.4044;700.4062;339.1732;138.1266 |
| 68 | 24.31 | [M+FA-H]^-^ | 1589.7269 | 2.6 | C_71_H_116_O_36_ | 1544.72 | Dipsacus saponin XII | / |
| 69 | 24.61 | [M-H]^-^ | 517.0997 | 1.8 | C_24_H_22_O_13_ | 518.11 | Emodin-8-O-( 6'-O-malonyl) -β-D-glucopyranoside | 473.1081;311.0527;269.0442;225.0548 |
| 70 | 24.93 | [M+FA-H]^-^ | 1101.435 | 5.9 | C_44_H_70_O_23_ | 966.43 | Rebaudioside A | 956.4229;803.3638 |
| 71 | 25.01 | [M+FA-H]^-^ | 849.3764 | 0.3 | C_38_H_60_O_18_ | 804.38 | Stevioside | 849.3858;803.3755;641.3202;479.2689 |
| 72 | 25.73 | [M-H]^-^ | 445.1141 | 0.2 | C_22_H_22_O_10_ | 446.12 | Physcion-8-β-D-glucoside | 329.2341;283.0597;240.0408 |
| 73 | 26.25 | [M+FA-H]^-^ | 973.5046 | 3.3 | C_47_H_76_O_18_ | 928.50 | Asperosaponin VI | 973.5084;927.4999;603.3915;323.0983;179.0565 |
| 74 | 27.61 | [M+H]^+^ | 191.1066 | -0.3 | C_12_H_14_O_2_ | 190.10 | Ligustilide | 191.1075;163.1120;149.0586;135.0436;115.0535 |
| 75 | 27.73 | [M+H]^+^ | 373.1284 | 0.6 | C_20_H_20_O_7_ | 372.12 | Isosinensetin | 373.1275;358.1083;343.0795;329.1069 |
| 76 | 28.22 | [M+FA-H]^-^ | 1015.519 | 7 | C_49_H_78_O_19_ | 970.51 | 4'-O-Acetylasperosaponin Ⅵ | 1015.5207;969.5115;645.4011;323.0988;179.0560 |
| 77 | 28.86 | [M+FA-H]^-^ | 1015.5191 | 7.1 | C_49_H_78_O_19_ | 970.51 | 2'-O-Acetylasperosaponin Ⅵ | 1015.5175;969.5092;645.4016;323.0984;179.0593 |
| 78 | 29.58 | [M+FA-H]^-^ | 1015.5143 | 2.3 | C_49_H_78_O_19_ | 970.51 | 3'-O-Acetylasperosaponin Ⅵ | 1015.5252;969.5120;645.4027;323.0987 |
| 79 | 29.69 | [M+FA-H]^-^ | 841.4597 | 0.7 | C_42_H_68_O_14_ | 796.46 | Dipsacussaponin A | 841.4592;795.4524;471.3474;323.0982 |
| 80 | 30 | [M+H]^+^ | 373.1284 | 0.6 | C_20_H_20_O_7_ | 372.12 | Sinensetin | 373.1282;343.0803;329.1045;312.0900 |
| 81 | 30.14 | [M+H]^+^ | 343.1178 | 0.5 | C_19_H_18_O_6_ | 342.11 | 4',5,7,8-Tetramethoxyflavone | 343.1231;313.0701 |
| 82 | 30.61 | [M+FA-H]^-^ | 957.5103 | 4 | C_47_H_76_O_17_ | 912.51 | Asperosaponin V | 957.5093;911.5039;587.3952;323.0959 |
| 83 | 32.48 | [M+H]^+^ | 403.1383 | -1.1 | C_21_H_22_O_8_ | 402.13 | Nobiletin | 403.1388;388.1164;373.0910;355.0830;327.0855 |
| 84 | 32.76 | [M-H]^-^ | 1351.6647 | 7.9 | C_64_H_104_O_30_ | 1352.66 | Dipsacus saponin C | / |
| 85 | 32.89 | [M-H]^-^ | 1219.6183 | 5.4 | C_59_H_96_O_26_ | 1220.62 | Dipsacus saponin B | 1219.6075;1073.5476;603.4041 |
| 86 | 33.61 | [M+H]^+^ | 193.1227 | 2 | C_12_H_16_O_2_ | 192.12 | Senkyunolide A | 151.9062;147.1167;129.0684;105.0698;91.0532 |
| 87 | 33.85 | [M+H]^+^ | 433.149 | -0.7 | C_22_H_24_O_9_ | 432.14 | 3',​4',​3,​5,​6,​7,​8-​Heptamethoxyflavone | 433.1490;418.1266;03.1028;385.0929;345.0574 |
| 88 | 34.59 | [M+H]^+^ | 373.1297 | 4.1 | C_20_H_20_O_7_ | 372.12 | Tangeretin | 373.1282;358.1052;343.0808;325.0694;297.0736;271.0604 |
| 89 | 35.93 | [M+FA-H]^-^ | 649.3965 | 1.2 | C_35_H_56_O_8_ | 604.40 | Cauloside A | 649.4001;603.3878 |
| 90 | 36.08 | [M+H]^+^ | 297.149 | 1.6 | C_19_H_20_O_3_ | 296.14 | Isocryptotanshinone | 297.1518;281.0516;253.1597;237.0914;211.1102 |
| 91 | 36.38 | [M-H]^-^ | 269.0455 | -0.2 | C_15_H_10_O_5_ | 270.05 | Emodin | 269.0444;241.0495;225.0553;197.0604 |
| 92 | 36.72 | [M+H]^+^ | 279.1023 | 2.6 | C_18_H_14_O_3_ | 278.09 | Dihydrotanshinone I | 279.0998;261.0944;233.0973;205.1032;189.0682 |
| 93 | 37.46 | [M+H]^+^ | 281.1178 | 2.1 | C_18_H_16_O_3_ | 280.11 | Trijuganone B | 281.0492;263.1086;235.1124;217.1013;202.0771;192.0932 |
| 94 | 38.44 | [M+H]^+^ | 520.3397 | -0.1 | C_26_H_50_NO_7_P | 519.33 | 1-Linoleoyl-sn-glycero-3-phosphocholine | 520.3419;502.3317;184.0733;104.1064 |
| 95 | 39.35 | [M+H]^+^ | 496.3402 | 0.9 | C_24_H_50_NO_7_P | 495.33 | 1-Palmitoyl-sn-glycero-3-phosphocholine | 496.3458;478.3329;184.0746;104.1071 |
| 96 | 39.63 | [M+H]^+^ | 297.1486 | 0.3 | C_19_H_20_O_3_ | 296.14 | Cryptotanshinone | 297.1481;279.1364;254.0942;251.1437 |
| 97 | 39.81 | [M+H]^+^ | 277.0851 | -3 | C_18_H_12_O_3_ | 276.08 | Tanshinone I | 277.0868;249.0898;203.0864 |
| 98 | 42.67 | [M+H]^+^ | 295.1319 | -3.3 | C_19_H_18_O_3_ | 294.13 | Tanshinone IIA | 295.1313;277.1243;266.0915;249.1295;234.1057;191.0852 |
| 99 | 46.48 | [M+H-H2O]^+^ | 439.3558 | -2.9 | C_30_H_48_O_3_ | 456.36 | Oleanolic acid | 439.3550;393.3543;289.2218;255.2142;247.1727;203.1804 |
